# Effectiveness of WeChat-group-based parental health education in preventing unintentional injuries among children aged 0–3: randomized controlled trial in Shanghai

**DOI:** 10.1186/s12889-022-14462-5

**Published:** 2022-11-16

**Authors:** Yuheng Feng, Xueqi Ma, Qi Zhang, Ruo Jiang, Jun Lu, Kaiyue Chen, Huiping Wang, Qinghua Xia, Jicui Zheng, Jingwei Xia, Xiaohong Li

**Affiliations:** 1 Department of Health Policy and Management, School of Public Health, Fudan University, 130 Dong’an Road, P.O. Box 177, Shanghai, 200032 China; 2 Research Center On Disability Issues, Fudan University, Shanghai, 200032 China; 3 Key Laboratory of Health Technology Assessment, National Health Commission, Fudan University, Shanghai, 200032 China; 4 School of Community and Environmental Health, Old Dominion University, Norfolk, VA 23529 USA; 5 Community Health Center of Jiading Town, Jiading District, Shanghai, China; 6 Changning District Center for Disease Control and Prevention, Shanghai, China; 7 Affiliated Pediatric Hospital of Fudan University, Shanghai, China; 8 Shanghai Huangpu District Maternal and Child Health Care Institute, Shanghai, China

**Keywords:** WeChat-based, Parents, Unintentional injury, Community-based, Health education intervention, Randomized controlled trial

## Abstract

**Background:**

Unintentional injuries to children are a major public health problem. The online social media is a potential way to implement health education for caregivers in online communities. Using WeChat, a free and popular social media service in China, this study evaluated the effectiveness of social online community-based parental health education in preventing unintentional injuries in children aged 0–3.

**Methods:**

We recruited 365 parents from two community health centers in Shanghai and allocated them into intervention and control groups randomly. Follow-up lasted for one year. The intervention group received and followed their WeChat group and a WeChat official account for dissemination of reliable medical information. The control group received only the WeChat group.

**Results:**

Between the intervention and control groups, changes in unintentional injuries (OR = 1.71, 95% CI: 1.02–2.87, *P* = *.*04), preventability (β = 0.344, 95% CI: 0.152–0.537, *P* < *.*001), daily supervision behavior (β = 0.503, 95% CI: 0.036–0.970, *P* = *.*04), and behaviors for preventing specific injuries (β = 2.198, 95% CI: 1.530–2.865, *P* < *.*001) were significantly different, and change in first-aid skills for treating a tracheal foreign body were nearly significant (*P* = *.*06).

**Conclusions:**

The WeChat-group-based parental health education can reduce the occurrence of unintentional child injuries by improving parents’ skills, beliefs, and behaviors. Online social communities promote health education and reduce unintentional injuries among children.

**Trial registration:**

ChiCTR1900020753. Registered on January 17, 2019.

**Supplementary Information:**

The online version contains supplementary material available at 10.1186/s12889-022-14462-5.

## Background

Unintentional injuries are a major public health threat to children worldwide, and they have become a key contributor to high disability and mortality rates [1]. In China, according to the *National Surveillance Data 2020*, unintentional injuries are the leading cause of death among children. For young children, most injuries occur at home [2–4]. The younger the child, the higher the occurrence of unintentional injuries [5].

Parents and other caregivers play an important role in the safety of young children [6, 7]. Evidence shows that the main reasons for unintentional injuries among young children at home were the parents’ lack of safety knowledge, their attitudes and behaviors, and the children’s attributes [8, 9]. Ma et al. found that improving parents’ knowledge, attitudes, and behaviors had a positive effect on unintentional injury occurrence in children and that attitudes also serve as mediators between knowledge and behaviors [10]. Although children aged 0–6 are a priority population under *The National Basic Public Health Service Program,* a systematic review has stated that a majority of studies focus on children aged 0–3 years [11].

To improve parents’ knowledge, attitudes/beliefs, and behaviors and guard child safety, various types of interventions have been made, such as education, safety devices, supervision, and community campaigns, to reduce child unintentional injury and improve caregivers’ knowledge, attitudes/beliefs, and behaviors [11, 12]. However, these implemented interventions have been offline, which wastes personnel hours and time and lacks sustainability. To address this problem, it is useful to explore the viability of online social media interventions through smartphones.

The utilization of smartphones is widespread in China, which offers opportunities for parent-based interventions. Smartphone-based applications have great potential for health intervention [13, 14]. Hu et al. developed a new application and implemented intervention with parents; however, developing and popularizing such interventions is expensive and difficult to generalize [15]. We need a cheap and generalized intervention. The World Health Organization has stated that community-based interventions can reduce child unintentional injuries effectively by using relevant information on local patterns [16]. A study generalized a community-based health education intervention to five counties in China to reduce environmental risk factors [17]. Communities often comprise people who have the same cultural background and aims [16].

However, due to the coronavirus disease 2019 (COVID-19) pandemic, traditional social interaction has been limited. Some studies have confirmed that interventions via social media are correlated with adolescents’ health [18, 19]. The online social community, which is a combination of online social media and real-life communities, can be established by a daily communication software and as an intervention platform, a pattern that is similar to traditional communities. Online social community-based intervention is also cost-effective [20].

In China, WeChat is a popular free social media service with about 576 million users as of 2017 [21, 22], which facilitates communication among parents and the establishment of online social groups. Hence, WeChat provides a convenient and promising platform to conduct health interventions. This study conducted a WeChat-group-based parental health education intervention on parents of children aged 0–3 and assessed its utility.

## Methods

### Conceptual framework

**Fig. 1:**
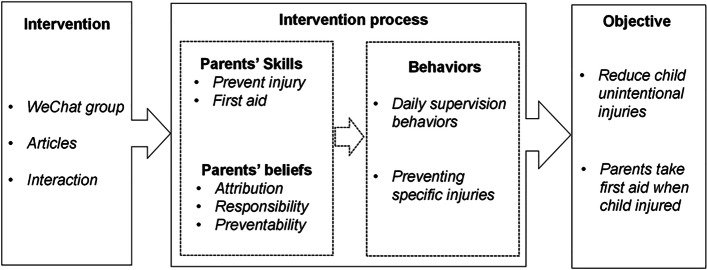
Conceptual framework

The health education intervention objectives of this study were reduced unintentional child injuries and increased use of first aid by parents when their children had injuries. To achieve these objectives, parents need to engage in appropriate behaviors, which are shaped by the cultivation and acquisition of relevant beliefs and skills. Behaviors can directly influence objectives, while skills and beliefs can indirectly impact objectives (Fig. 1).

In accordance with the literature on the behaviors of parents and children toward unintentional injury prevention [23–25], this study divided behaviors into daily supervision behaviors to prevent specific injuries and appropriate reactive behaviors in the form of proper first-aid provision in the event of child injury.

As preventive behaviors entail daily parental supervision—which means that parents care for, supervise, and focus their attention on their children’s actions to avoid specific injuries—parents have to possess appropriate and adequate knowledge of the measures to avert such injuries. For example, they should know how to prepare bathwater for their children to avoid burns.

In accordance with the literature on parents’ beliefs concerning unintentional child injury [26], certain locus of control theories [27], the big five model [28], and the health beliefs model [29, 30], we divided beliefs into attribution, preventability, and responsibility beliefs. “Attribution beliefs” refer to parents’ cognition regarding the reasons for and causes of unintentional injuries, “responsibility beliefs” denote whether they recognize their obligation to care for and supervise their children, and “preventability beliefs” indicate parents’ cognition about what they need to do to protect their children from injuries. “Skills” imply parents’ ability to prevent and mitigate unintentional injuries, for example by taking measures, including making changes to the home environment and applying first aid such as cardio-pulmonary resuscitation. Based on these measures, this study considered parents to be direct intervention participants and evaluated the effectiveness of ensuring/improving these parental attributes on unintended child injury.

### Intervention tool

Parents of young children need to possess and portray certain skills and behaviors to ensure the safety of their children, which means that they may have to be taught these behaviors and skills [10]. This study is based on the Haddon matrix tool [31], which suggests that any accident is affected by a host, an agent, and the environment. The matrix analyzes the interaction of these elements according to the time around the injury—pre-, during, and post-injury [32]. Accordingly, this study first considered various injuries that children aged 0–3 may experience; according to severity and susceptibility, they were categorized via expert argumentation. Eventually, five kinds of injuries were identified and an intervention tool, in the form of educational studies, was designed. Second, to safeguard the quality of the studies, parents’ beliefs were considered comprehensively. As the intervention was based on the WeChat platform, it included only articles that were released on our WeChat official account and delivered via the WeChat group. We finalized 30 studies (Supplementary Fig. 1) based on the aforementioned categories; the list is provided in Supplementary Table 1.

### Study design

This study conducted a two-center, parallel, participant-blind randomized controlled trial of a WeChat-group-based parental health education intervention from March 2019 to March 2020 to evaluate the effectiveness of the health education intervention. Co-author Jiang was responsible for generating the random allocation sequence and the community health center enrolled participants through objective sampling and assigned them to either intervention group or control group. One-on-one interviews were conducted by the investigators, who were members of the research group and had received training. During the investigation process, quality control personnel checked the completed items. This study did not have an important change in its methods after trial commencement. It was implemented and reported based on the Consolidated Standards of Reporting Trials (CONSORT) 2010 statement [33].

### Data collection

A questionnaire comprising items on socio-demographic characteristics and the skills required to respond to, beliefs about, and behaviors toward unintentional injuries was used for data collection. The skill component consisted of a question about aid measures. The beliefs component covered injury attribution, which was measured by a single item: “It is owing to bad luck that children get unintentionally injured.” The responsibility and preventability component was evaluated using two questions. The behaviors component covered daily supervision behaviors, which were assessed via five questions, and behaviors to prevent specific injuries, which were measured via items on several different injuries drawn from Ma et al.’s research [10]. Supplementary Table 2 outlines the questionnaire items. The Cronbach’s α of beliefs was 0.76.

For community childcare doctors, we conducted an in-depth interview to understand their feelings and opinions during and after the intervention, and their suggestions about extending the intervention.

### Participants

Parents dedicated to regular child health examination were recruited from January 1 to February 28, 2019 in two community health centers in Jiading Town Street and Juyuan New District, respectively, as the sample area comprising 38 resident committees. This area is located in the northwest of Shanghai and covers an area of 464.2 km^2^. Shanghai’s annual per capital gross domestic product (GDP) in 2018 was 134,982 yuan, and ranked second among the 31 provinces of mainland China [34]. In 2017, Jiading District’s annual per capita GDP was 104,423 yuan, thus ranking sixth among Shanghai’s 16 districts. Approximately 3,000 children aged 0–3 live in the central area of Jiading District [35].

The selection of parents followed specific inclusion and exclusion criteria. The inclusion criteria were parents (1) of children aged 0–3, who went to the community health center for regular physical examinations, (2) whose children were local residents and registered in the local community health center, (3) who could read, and (4) who voluntarily provided written informed consent to participate in this study.

The exclusion criteria were parents (1) who could not participate in the face-to-face interviews, (2) whose child had a disability, (3) whose child was one of twins or multiples, (4) who could not read, (5) who were not accompanying the child, and (6) who were unwilling or unable to sign an informed consent form.

### Patient and public involvement

Patients or the public were not involved in the design, conduct, reporting or dissemination plans of our study.

### Sample size calculation

We calculated the sample size based on the formula for comparing two occurrence rates with at a 1:1 ratio in the intervention and control groups. A non-controlled intervention study in China demonstrated that unintentional injury rates before and after intervention were 23% and 4%, respectively [36]. The participants were children aged 1–6, and the procedure included on-site inspections and risk factor modifications in home environments. Our study was based on an online intervention and supposed that the injury rate of the control group after one year is 23% and that of the intervention group is 10%. We determined that the sample size of the intervention and control groups should be 125 people. Assuming a dropout rate of 10%–50% (based on typical rates for online interventions) [37, 38], 138–188 parents were required for each group. All methods were performed in accordance with relevant guidelines and regulations.

## Randomization

A total of 365 participants were enrolled. We stratified parents based on children’s age and encoded parents according to random number table via SPSS 26.0 in each layer. Finally, they were divided between intervention and control group randomly at a 1:1 ratio: 182 were placed in the intervention group and 183 in the control group.

## Intervention component

The health education intervention program focused on helping parents become aware of the possibility and severity of potential child injuries, improve their knowledge/beliefs about and skills required to respond to unintentional child injuries, and develop supervision behavior. The intervention consisted of a WeChat group and WeChat official account named “children safety and health” (Supplementary Fig. 2).

### Intervention group

First, the WeChat official account was registered and designed. Based on books on unintentional injury prevention in children, 30 studies, which were reviewed by the community childcare doctors, were produced and released. The title length of each study was about 20 words and content length, which included text and pictures, was limited to about 400 words. Second, we developed 30 text messages based on the 30 studies; each included key information from the corresponding study. Third, the WeChat group was established. It comprised the parents, physicians from the community health center, and an assistant, a graduate student from the author’s university.

Health education intervention conduction precedence and delivery were as follows. (1) The assistant of the community childcare doctor uploaded one of the 30 studies to the WeChat official account and sent a text message and the link to the WeChat groups at about 7:00 p.m. every week during the first three months. To reflect the real-world setting, the text messages to the WeChat group were only delivered as notifications, and it was not mandatory for parents to read them. (2) Parents in the intervention group registered on the WeChat official account so they could access the studies on that. (3) Parents could send messages to the WeChat group if they had any questions, and the community childcare doctor in the WeChat group was required to respond within 48 h. All the group members could interact with each other (Supplementary Fig. 3). Links to the studies were sent to the WeChat group. (4) A few first-aid videos were transmitted from official websites or WeChat official accounts.

### Control group

The assistant of the community childcare doctor established this group; however, neither the assistant nor the doctor sent any information about unintentional injury prevention. Additionally, the control group did not follow the WeChat official account.

### Outcome measures

#### Primary outcome

The primary outcome was child unintentional injury occurrence rate during the past one year, which was calculated according to the number of children who were injured divided by their total number. An injury event is defined as an event that meets any of the following criteria by the International Classification of Diseases, 10th Revision: (1) the child received medical treatment by a doctor or another medical professional after being injured; (2) the child received first aid such as medication, massage, or a hot compress from a member of the family, a teacher, or other non-medical personnel after being injured; (3) the child could not attend school or participate in other activities, was bedridden, or rested for more than half a day after the injury [39]. Owing to the common occurrence of child injuries and their differing degrees, the injuries in this study were limited to those requiring medical attention.

#### Secondary outcome

The secondary outcomes were response skills, which were mainly related to first aid for a tracheal foreign body, beliefs about injury attribution, responsibility and preventability, daily supervision behaviors, and behaviors for preventing specific injuries. They were measured using the questionnaire described earlier.

### Statistical analysis

Regarding the socio-demographic data, this study transformed continuous data such as children’s age, fathers’ age, and mothers’ age, into category data, which had a grade meaning. For fathers’ and mothers’ occupations, in accordance with China’s statistical yearbook, we divided the data into four groups—employees of state-owned enterprises and public institutions, employees of foreign-funded and private enterprises, other (farmers, soldiers, freelancers), and unemployed, which did not represent grade meaning. Then, the socio-demographic characteristics were described based on frequencies and percentages (n [%]). To evaluate the differences in the frequency of reading the WeChat group messages and the helpfulness of interacting with other parents between intervention and control group after the follow-up and the acceptance of the WeChat group, the chi-squared and Wilcoxon rank-sum tests were performed.

For an effective outcome that included both primary and secondary outcomes, we described them via mean and 95% confidence intervals (95% CIs) in the baseline and follow-up data. The expectation–maximization algorithm was used to input the missing values. To evaluate whether differences between intervention and control group existed, the generalized estimation equation (GEE) was used. During the GEE analysis process, as child unintentional injury occurrence rate is a binary variable, it is calculated by binary logistic regression model that the indicator is the odds ratio (OR). OR means the function of the intervention program: if OR > 1, the intervention can reduce child unintentional injury effectively. The result of OR minus one indicated what extent the changes in child unintentional injury occurrence rate can be attributed to the intervention program. Additionally, as beliefs and behaviors are continuous variables, they are calculated by a linear model in which the indicator is beta (β). The value of β indicates the mean amount of change in the total score of beliefs and behaviors when an independent variable changes one unit (received intervention) and the other independent variables remain constant. Statistical tests were two-sided, and a *p*-value < 0.05 indicated significance.

## Results

### Socio-demographic characteristics

**Fig. 2:**
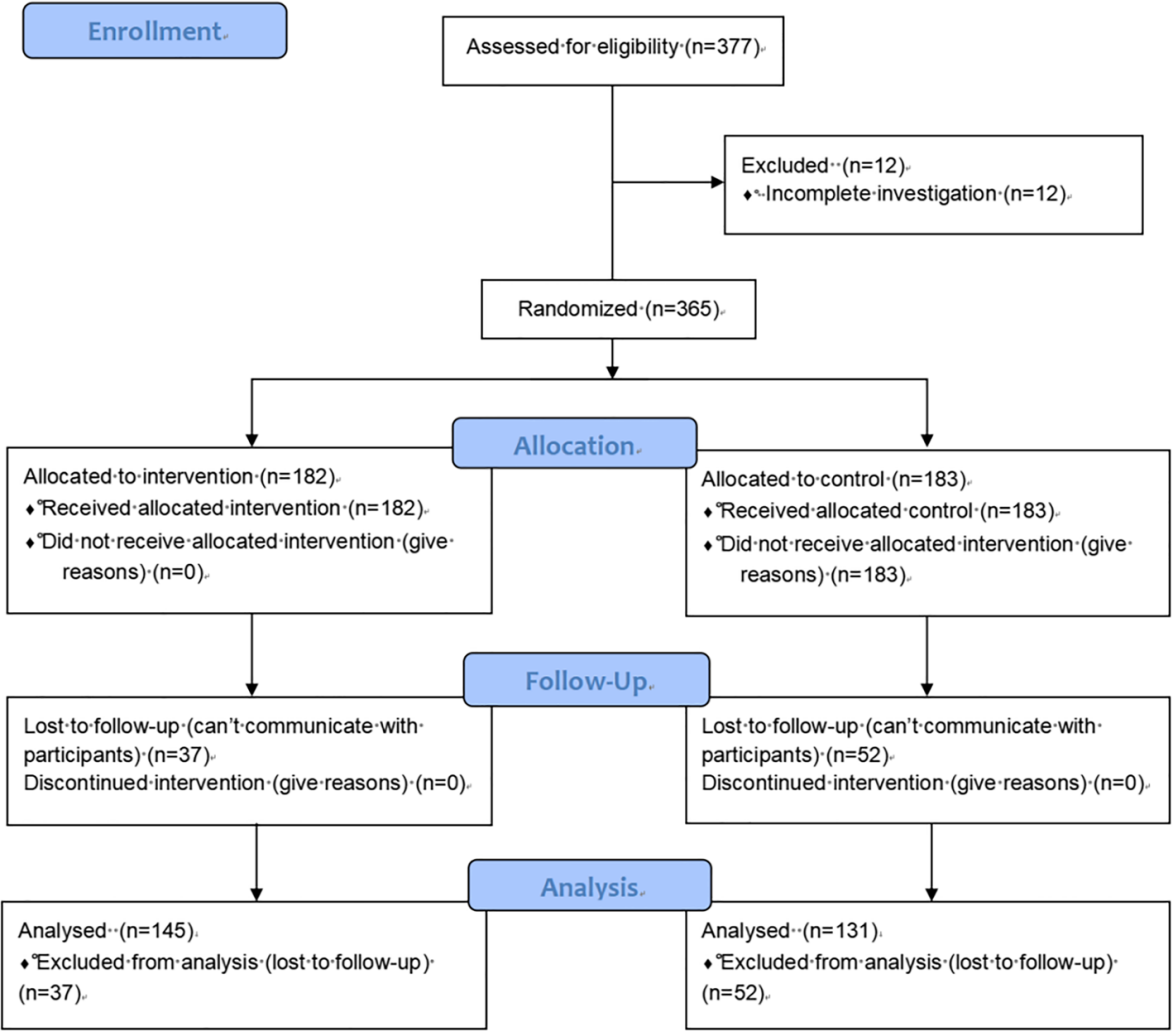
Study flow diagram

At baseline, 365 participants were included; eventually 276 participants completed the trial. Figure 2 presents the flow diagram.

This study compared the socio-demographic characteristics between intervention group and control group to determine whether there was homogeneity between the participants. The results were non-significant (*P* > 0.05; Table 1).

**Table 1:** Socio-demographic characteristics of sample, comparing intervention group with control group

Variable	Intervention group *n* = 145	Control group *n* = 131	*P*
Children’s age (years), n (%) ^a^			.94
0–	54 (37.2)	49 (37.4)	
1–	46 (31.7)	40 (30.5)	
2–	45 (31.0)	42 (32.1)	
Fathers’ age (years), n (%) ^a^			.35
< 25	8 (5.5)	4 (3.1)	
25–30	49 (32.4)	38 (29.0)	
30–35	57 (38.6)	56 (42.7)	
> 35	35 (23.5)	34 (25.2)	
Fathers’ education, n (%) ^a^			.09
Senior high school or below	14 (9.7)	14 (10.7)	
College	36 (24.8)	17 (13.0)	
Undergraduate or above	95 (65.5)	100 (76.3)	
Fathers’ occupation, n (%) ^b^			.12
Employee of state-owned enterprises and public institutions	43 (29.7)	47 (35.9)	
Employee of foreign-funded, private and enterprises	67 (46.2)	65 (49.6)	
Other (farmers, soldiers, freelancers)	35 (24.1)	19 (14.5)	
Unemployed	0 (0)	0 (0)	
Mothers’ age (years), n (%) ^a^			.05
< 25	15 (10.3)	9 (6.9)	
25–30	68 (46.9)	49 (37.4)	
30–35	41 (28.3)	51 (38.9)	
> 35	21 (14.5)	22 (16.8)	
Mothers’ education, n (%) ^a^			.18
Senior high school or below	19 (13.1)	16 (12.2)	
College	33 (22.8)	20 (15.3)	
Undergraduate or above	93 (64.1)	95 (72.5)	
Mothers’ occupation, n (%) ^b^			.17
Employee of state-owned enterprises and public institutions	34 (23.4)	36 (27.5)	
Employee of foreign-funded, private and enterprises	54 (37.2)	60 (45.8)	
Other (farmers, soldiers, freelancers)	42 (29.0)	27 (20.6)	
Unemployed	15 (10.3)	69 (25.0)	
Score of knowledge, mean (95% CI) ^c^	4.41 (4.19–4.63)	4.50 (4.26–4.75)	.56

Abbreviations: *CI* confidence interval

^a^ For age and education, we used the Wilcoxon rank-sum test between groups

^b^ For occupation, we analyzed chi-squared tests groups

^c^ For knowledge score, we analyzed data with two independent t-tests between groups

### Implementation and acceptance of the WeChat group

During the interview, community childcare doctors deemed the WeChat-based health education intervention effective and determined that it did not impose too much workload on the doctors. The intervention only required them to spend an average of 10 min answering parents’ questions per day. They stated that nurses were qualified and best suited for answering parents’ questions, which could enhance the health education intervention effectiveness greatly. Their feedback demonstrated that the WeChat group can also serve as an information source and a health education platform for other parents outside of WeChat and that this intervention was an efficient and safe online method to use, especially during the ongoing COVID-19 pandemic. For the economic burden of disease, unintentional injuries led to not only direct but also indirect medical expenses such as parents needing to spend at least half a day taking children to hospital.

The results showed that the proportion of parents “often” and “always” reading the WeChat group messages was 68.1%. The intervention group checked the WeChat group messages more frequently than the control group (*P* = *0.0*2). Parents in both groups believed that the information in the WeChat group was helpful (*P* = *0.7*1). Of the intervention group parents, 84.1% deemed the studies and text messages helpful, 86.9% reported that the doctors’ online answers in the WeChat group were helpful, and 68.0% responded that they would “often” or “always” teach other family members about preventing unintentional child injuries after acquiring the knowledge (Supplementary Table 3).

### Unintentional injury occurrence rate

For the intervention group, the unintentional injury occurrence rate at baseline was 9.0% and at follow-up was 11.7%, which was not significant (*P* = *0.4*3). For the control group, the unintentional injury occurrence rate at baseline was 9.9% and at follow-up was 22.9%, which was significant (*P* = *0.0*04). The difference between the intervention and control group was significant (OR = 1.71, 95% CI: 1.02–2.87, *P* = *0.0*4).

### Parents’ skills, beliefs, and behaviors

For each group, the number of participants included in each analysis were analyzed by original assigned groups.

Regarding parents’ skills, comparing the intervention and control group, the difference on first aid for tracheal foreign body was nearly significant (*P* = *0.0*6). Regarding parents’ injury attribution, responsibility, and preventability, in the intervention group, the scores were 3.46, 6.60, and 6.92 at baseline; and 3.59, 7.52, and 7.72 at follow-up, respectively; changes were significant (*P* = *0.0*07, *P* < *0.0*01, and *P* < *0.0*01, respectively). In the control group, the scores were 3.49, 6.80, and 7.02 at baseline; and 3.38, 6.99, and 6.94 at follow-up, respectively. These changes showed statistical significance for injury attribution (*P* = *0.0*4) but no statistical significance for responsibility and preventability (*P* = *0.0*7 and P = 0.33, respectively). Comparing the intervention and control groups, the differences were not significant for injury attribution (β = 0.091, 95% CI: -0.028 to 0.210, *P* = *0.1*3) and responsibility (β = 0.165, 95% CI: -0.014 to 0.345, *P* = *0.0*7) but were significant for preventability (β = 0.344, 95% CI: 0.152–0.537, *P* < *0.0*01).

**Fig. 3:**
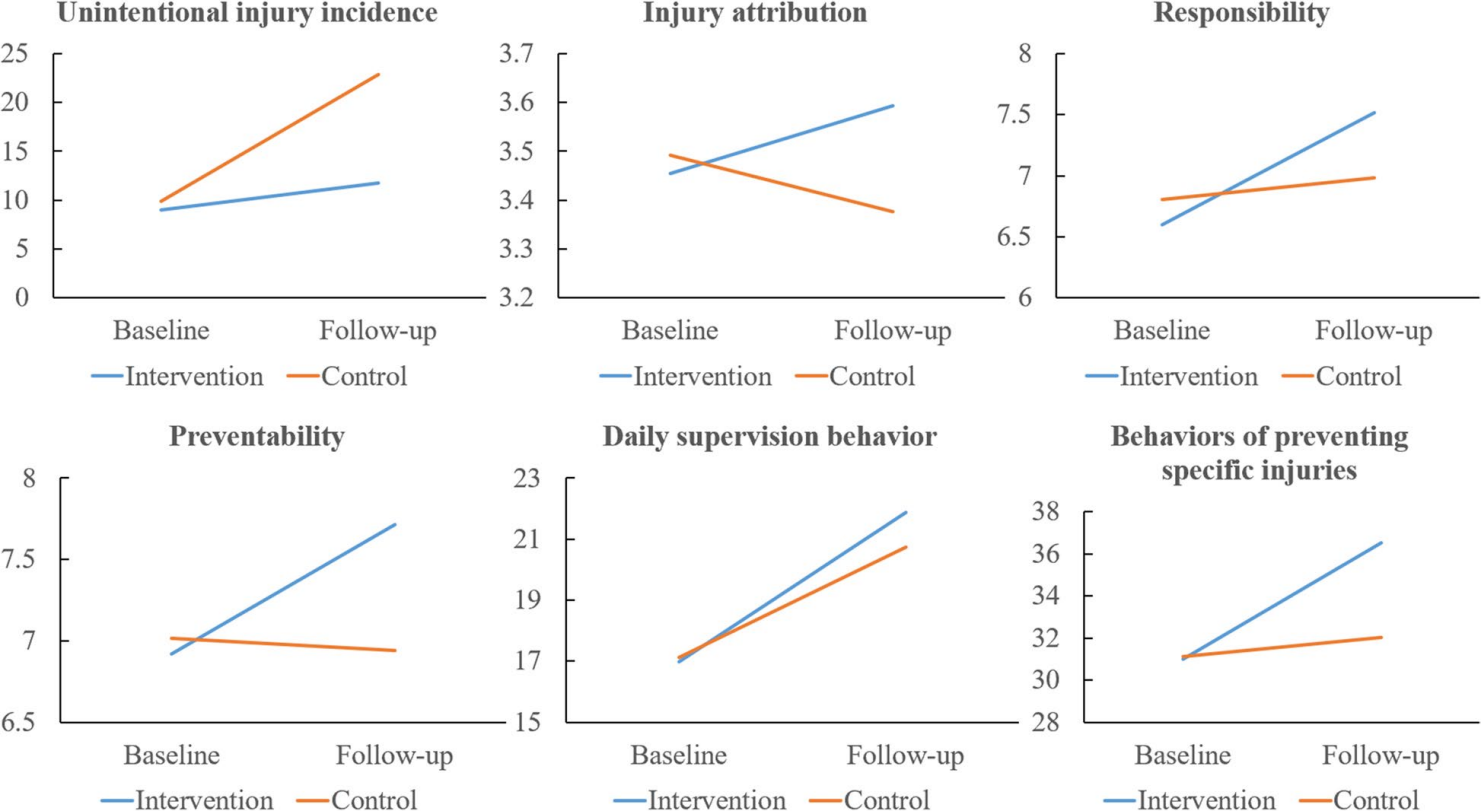
Comparison between baseline and follow-up of the intervention and control groups

For parents’ daily supervision behavior and behaviors for preventing specific injuries, in intervention group, the scores were 16.99 and 30.99 at baseline and 21.88 and 36.53 at follow-up, respectively (*P* < *0.0*01 and *P* < *0.0*01, respectively). In the control group, the scores were 17.12 and 31.10 at baseline and 20.75 and 32.02 at follow-up, respectively (*P* = *0.0*4 and *P* < *0.0*01, respectively). Comparing the intervention and control group, the differences were significant for daily supervision behaviors (β = 0.503, 95% CI: 0.036–0.970, *P* = *0.0*4) and behaviors for preventing specific injuries (β = 2.918, 95% CI: 1.530– 2.865, *P* < *0.0*01; Table 2, Fig. 3, and Supplementary Table 4).

**Table 2:** The effectiveness of primary and secondary outcome

Variable	Intervention group (*n* = 145)		control group (*n* = 131)		OR ^a^ or β ^b^	*P* ^c^
	Baseline (95% CI)	Follow-up (95% CI)	Baseline (95% CI)	Follow-up (95% CI)		
Unintentional injury occurrence rate, %	9.0 (4.1, 13.9)	11.7 (6.2, 17.2)	9.9 (4.8, 15.0)	22.9 (15.7, 30.1)	1.71 (1.02, 2.87)^a^	.04
First aid for tracheal foreign body, % ^d^	—	70.3 (62.9, 77.8)	—	80.2 (73.3, 87.0)	—	.06
Injury attribution	3.46 (3.36, 3.55)	3.59 (3.50, 3.69)	3.49 (3.40, 3.58)	3.38 (3.26, 3.49)	0.091 (-0.028, 0.210)^b^	.13
Responsibility	6.60 (6.45, 6.76)	7.52 (7.40, 7.63)	6.80 (6.62, 6.98)	6.99 (6.82, 7.15)	0.165 (-0.014, 0.345)^b^	.07
Preventability	6.92 (6.75, 7.08)	7.72 (7.62, 7.81)	7.02 (6.83, 7.20)	6.94 (6.77, 7.11)	0.344 (0.152, 0.537)^b^	< .001
Daily supervision behavior	16.99 (16.74, 17.24)	21.88 (21.58, 22.18)	17.12 (16.91, 17.33)	20.75 (19.92, 21.58)	0.503 (0.036, 0.970)^b^	.04
Behaviors of preventing specific injuries	30.99 (30.37, 31.60)	36.53 (36.16, 36.91)	31.10 (30.49, 31.71)	32.02 (31.44, 32.61)	2.198 (1.530, 2.865)^b^	< .001

Abbreviations: *CI* confidence interval, *OR* odds ratio. The statistical difference compared SKG and KG was argued by 95% CI whether contained “0”

^a^ The indicator is OR. For unintentional injury occurrence rate, we calculated OR, which equaled exp (β)

^b^ The indicator is β, non-standardized regression analysis parameter estimates adjusted according to child age

^c^ This section was analyzed by GEE model to state whether intervention had an impact on outcomes

^d^ This section was analyzed by Chi-squared test between intervention and control group for follow-up data

## Discussion

This study is the first to examine the effectiveness of a WeChat-group-based health education intervention to prevent unintentional injuries among Chinese children aged 0–3 by improving parents’ skills, beliefs, and behaviors toward such injuries. The results suggest that the health education intervention had a positive influence on reducing child unintentional injury occurrence rate, and enhancing parents’ skills, beliefs about preventability, and behaviors. However, no significant difference was found concerning beliefs about injury attribution and responsibility between the intervention group and control groups. The health education intervention also had a specific impact on parents’ skills, thus leading to improved parent behavior. This result is consistent with that of Ma et al., which showed that children directly affected parental behavior, which directly influenced preventability [10]. These factors form a mechanism that reduces child unintentional injuries. As children grow, they become exposed to different risks that could lead to unintentional injuries if parents do not take measures to prevent them, which could result in increases in injury occurrence rate. Therefore, an effective health education program such as the one proposed here is necessary.

For injury attribution, a study suggests that unintentional injury is worth fighting against to avoid fatalities [40]. Though the goal of this study was to contribute to the prevention of unintentional child injuries, certain types of beliefs were difficult to alter [41], and the changes that occurred were not significant after the health education, which suggest that some personal characteristics are not easily reversed. In future research, the health education intervention should be provided according to different injury attributions and the aim should be to improve parents’ understanding on the topic.

Based on the baseline data, the scores of parents’ knowledge about unintentional injury were not significant between the intervention and control groups, which is relatively ideal. The parents were deemed to have portrayed responsibility as they expressed willingness to receive education about unintentional child injury prevention.

The current trial result was similar to that of Wang et al. [42], who used safety promotion to help mothers of toddlers via health education, social support, and target setting based on WeChat, and demonstrated that safety promotion had the potential to promote toddlers’ safety at home. Thus, the focus of this study can change children’s environments by enhancing parents’ and other caregivers’ safety awareness.

Although a few studies reported that environmental changes reduced child injuries, a systematic review demonstrated that the evidence was limited and that there was considerable proof that environmental changes could reduce home hazards [43]. Our study focused on children aged 0–3, who were mainly cared for by parents and other caregivers and live at home. It provided strong evidence to verify that environmental changes can reduce unintentional child injuries. In China, community-based basic public services with wide coverage have been fully implemented [44], and communities provide health management services for children aged 0–6 [45]. Basic public health services can be implemented in areas with similar demographic and economic characteristics. The WeChat-group-based health education proposed here was similar to the real-world setting; therefore, both parents and healthcare providers were ready to accept it. The results supported the promotion of WeChat-group-based health education centered on community-based basic public services. In addition, as social applications are increasingly emerging in other countries that are familiar with WeChat, this study also provided practice evidence. If these applications can be used to implement intervention, medical expenses and economic burden can be saved. In China, the economic burden to society owing to child injuries is about 10.86–45.33 billion yuan annually, including 3.26 billion yuan in medical expenses [46].

As the WeChat app is free and widely used in daily life in China, both the cost of developing the application and the cost of training the parents and the doctors to get familiar with the application were negligible. The only cost was that of consulting time for doctors who answered the questions of parents in the WeChat group. According to China’s healthcare human resource statistics, of the approximately 260 workdays in a year, community childcare doctors spent only about 3,000 min answering parents’ questions; thus, their salary per minute increased from 1.1 to 1.2 yuan [47]. Therefore, this study indicated that the cost of the one-year intervention was 3,300–3,600 yuan, less than half of the annual per capita disposable income of urban residents, which was 76,437 yuan in 2020 according to the National Bureau of Statistics. The average direct medical cost of outpatient care for unintentional injuries among children aged 0–6 was 115 yuan in Gansu province, located in western China [48]. In addition, according to a study on non-fatal unintentional injury among children 0–6 in Guangdong province, with similar economic status as Shanghai, the invisible loss was about three times the direct medical cost [49]. Considering the invisible loss owing to injury, the social loss owing to each outpatient of child unintentional injury was about 460 yuan.

After follow-up, 17 children in the intervention group incurred unintentional injuries, of whom 5 children were outpatients, while 30 children incurred unintentional injuries in the control group, and 9 went to hospital (8 children were outpatients and 1 child was an inpatient). If the occurrence of unintentional injury in the intervention group was 22.9%, 33 children incurred unintentional injuries and 10 children went to hospital (9 children were outpatients and 1 child was an inpatient). We deemed that the intervention measure avoided 16 children’s unintentional injuries and prevented 4 children from being outpatients and 1 child from being an inpatient. For outpatients, mere direct medical expenses could be saved in the amount of 460 yuan, while 1380 yuan could be saved if invisible loss was considered. Based on a previous survey, 41 participating children were injured and 13 children of them went to hospital (3 children were inpatients), and for inpatient, the total medical expenses were 15,000 and the per capita medical expenses were 5,000 [10]. For indirect medical expenses, parents could avoid about 106 yuan (76,437 / 360 / 2 = 106 yuan) in costs owing to the time spent in taking their children to hospital. For five children, the indirect medical expenses were 530 yuan. Thus, the intervention measure produced economic benefits (460 + 530 + 15,000 + 1,380 = 17,370 yuan) of 4.83–5.26 times the cost (3,300–3,600 yuan). Therefore, the health education intervention was economically sound.

This study had a few limitations. First, the questionnaires were completed by parents, which potentially led to social desirability bias. Parents may also deliberately conceal information, thereby affecting the results. Second, owing to ethical concerns, blank control is not suitable for this study; by comparing an intervention group with a control group, intervention effectiveness will be underestimated. Third, this study was conducted only in Jiading District, Shanghai. Parents there generally possess a higher education level than elsewhere in the country. Thus, generalizability is limited. Fourth, it is impossible to distinguish which played a more important role in preventing unintentional injuries—the health education provided by the WeChat official account or the interaction of the physician in the WeChat group. Fifth, the intervention is an online intervention program; thus, some parents may withdraw from follow-up for various reasons after receiving the whole intervention. Approximately 24% of parents were lost at follow-up. To further understand their reasons for withdrawing, the research team communicated with them by phone, and they stated that they were concerned with child information privacy.

Thus far, the proposed health education has been effective; however, the specific roles of the informative studies, WeChat group discussions, and doctor answers are unclear. Process analyses are needed in the future. Furthermore, future studies should focus on how to incentivize doctors to perform active online interventions.

## Conclusions

This WeChat-group-based health education intervention with parents of young children used WeChat as a platform to assess the effectiveness of a parent-centered online-based intervention against unintentional children’s injuries. The intervention group participants followed the WeChat official account and communicated with the community childcare doctors and other parents via a WeChat group. In contrast, the control group participants could not follow the official account; they communicated only with other parents and the community childcare doctor’s assistant on a WeChat group. After the intervention, we assessed the outcomes and compared them with baseline results. Additionally, we compared the data between the two groups. Our health education intervention had a positive influence on reducing unintentional injury occurrence and improving parents’ skills, beliefs, and behaviors. Online social community is worth promoting as a way of health education to improve the situation of unintentional injuries in children, as it can not only shorten social distance and save time and money when parents have emergency childcare questions but also enhance parents’ beliefs, skills, and behaviors by helping them communicate with other parents and the community childcare doctor frequently and quickly. Parents even can acquire additional relevant knowledge beyond that directly related to the health education intervention. In addition, communicating with others in via social media has become a daily habit in the current era; therefore, online social communities are a natural platform to implement health education interventions.

## Supplementary Information

**Additional file 1:** **Figure S1.** Design the articles based on Haddon model and beliefs the parents needed.

**Additional file 2:** **Figure S2.** Articles uploaded to the WeChat officialaccount.

**Additional file 3:** **Figure S3.** Screenshots of parents and doctorscommunicating with each other in the WeChat group.

**Additional file 4:** **Table S1.** Theclassification and focus of the 30 articles.

**Additional file 5:** **Table S2.** The details of questionnaire.

**Additional file 6:** **Table S3.** Acceptance of WeChat among intervention group and control group.

**Additional file 7:** **Table S4.** Theeffectiveness of primary and secondary outcome within each group.

## Data Availability

Data are available upon reasonable request. Xiaohong Li, the corresponding author, can be contacted.

## Abbreviations

CI: Confidence interval
GDP: Gross domestic product
GEE: Generalized estimation equation
OR: Odds ratio
