# Supplementary material for: Effectiveness of WeChat-group-based parental health education in preventing unintentional injuries among children aged 0–3: randomized controlled trial in Shanghai
Source: BMC Public Health. 2022 Nov 16;22:2086. doi: 10.1186/s12889-022-14462-5 (PMC9666943; doi:10.1186/s12889-022-14462-5)
Supplement: Supplementary file 1 — Additional file 1: Figure S1. Design the articles based on Haddon model and beliefs the parents needed. [file 12889_2022_14462_MOESM1_ESM.docx]

**Table S1.** The classification and focus of the 30 articles

| Classification | Articles | Teaching effect index | |
| --- | --- | --- | --- |
|  |  | Skills | Awareness |
| Falls |  |  |  |
|  | How to prevent babies aged 0-3 from falling, here's the full answer! | ++ | ++ |
|  | Tips for preventing falls in children. | ++ | ++ |
|  | You can do more to prevent your baby from falling | ++ | ++ |
| Burns |  |  |  |
|  | Put cold water or hot water first when bathing your baby? Unexpectedly, the correct way is.... | +++ | +++ |
|  | Here's the correct way to treat children's burns, come and check. | +++ | + |
|  | When parents give the baby a bath, put hot water first easy to burn, the correct approach is...... | ++ | +++ |
|  | How does darling burn do? Here's the answer! | +++ | + |
|  | A moment of negligence breeds tragedy! What parents need to know to prevent children from burning. | + | +++ |
| Drowning |  |  |  |
|  | Water is a source of life, but it can also rob people of their lives: prevent drowning in children, keep this in mind! | ++ | +++ |
|  | Does drowning happen in your home? The result was...... | + | +++ |
|  | A safe and happy swimming guide for children aged 0-3. | +++ | +++ |
|  | Preventing children from drowning at home, what parents have to know? | ++ | +++ |
|  | Children drowning first aid knowledge get up ~ | +++ | +++ |
| Poisoning |  |  |  |
|  | "Keep it high, keep it away from children"--small lecture hall for children poisoning prevention | ++ | +++ |
|  | Tips for preventing poisoning in children. | +++ | +++ |
|  | Alert! Remember these tips to save your baby's life when he gets poisoned | +++ | +++ |
| Asphyxia |  |  |  |
|  | Babies always choke water/milk, what should parents do? Here are a few ways to help you solve it easily | +++ | +++ |
|  | Children are prevented from choking before the age of 1. | ++ | +++ |
|  | 10 “not do” parents should pay attention to prevent baby asphyxia | ++ | +++ |
|  | First aid advice: Is it correct to pat children directly on the back for foreign body asphyxia? | +++ | ++ |
|  | Is your home also a child asphyxia hotspot? Prevention of suffocation at home can not be ignored! | + | +++ |
| Other |  |  |  |
|  | When buying things for your baby, pay attention to these! | + | +++ |
|  | Should babies sleep with their parents? | ++ | +++ |
|  | Do you know? There are invisible killers in your home. | + | +++ |
|  | Real cases of injuries in children under one year of age, parents must pay attention. | + | +++ |
|  | Parents should be the “safe umbrella” suitable for children's age. | ++ | +++ |
|  | When taking care of children, your eyes must not leave the children, even for a minute! | ++ | +++ |
|  | Children happen ankle sprain, should rest or massage? Cold compress or hot compress? | +++ | ++ |
|  | Parents must know: children happened trauma how to deal with! | +++ | +++ |
|  | Unintentional injuries to children frequently occur in summer, parents need to be aware. | + | +++ |
