# Supplementary material for: Effectiveness of WeChat-group-based parental health education in preventing unintentional injuries among children aged 0–3: randomized controlled trial in Shanghai
Source: BMC Public Health. 2022 Nov 16;22:2086. doi: 10.1186/s12889-022-14462-5 (PMC9666943; doi:10.1186/s12889-022-14462-5)
Supplement: Supplementary file 4 — Additional file 4: Table S1. Theclassification and focus of the 30 articles. [file 12889_2022_14462_MOESM4_ESM.docx]

**Table S3.** Acceptance of WeChat among intervention group and control group

| Variable | Intervention group  n=145 (%) | Control group  n=131 (%) | *P* value |
| --- | --- | --- | --- |
| The frequency of checking WeChat group messages ^a^ |  |  | 0.02 |
| Never | 3 (2.1) | 2 (1.5) |  |
| Seldom | 5(3.4) | 14 (10.7) |  |
| Sometimes | 27(18.6) | 37 (28.2) |  |
| Often | 57 (39.3) | 38 (29.0) |  |
| Always | 53 (36.6) | 40 (30.5) |  |
| Considering the interaction between parents in WeChat group were helpful ^b^ |  |  | 0.71 |
| Yes | 122 (84.1) | 108 (82.4) |  |
| No | 15.9 (23) | 23 (17.6) |  |
| Considering the 36 articles and text messages about unintentional injuries in WeChat group were helpful |  |  | — |
| Yes | 122 (84.1) | — |  |
| No | 23 (15.9) | — |  |
| Considering the doctor's online answers in the WeChat group were helpful |  |  | — |
| Yes | 126 (86.9) | — |  |
| No | 19 (13.1) | — |  |
| The frequency of teaching other family members after acquiring the knowledge of preventing children unintentional injuries |  |  | — |
| Never | 0 (0) | — |  |
| Seldom | 8 (5.3) | — |  |
| Sometimes | 40 (26.7) | — |  |
| Often | 36 (24.0) | — |  |
| Always | 66 (44.0) | — |  |

“The frequency of checking WeChat group messages” was analyzed by Wilcoxon rank sum test between intervention group and control group.

“Considering the interaction between parents in WeChat group were helpful” was analyzed by Chi-square test between intervention group and control group.
