# Supplementary figures and images for: Effectiveness of WeChat-group-based parental health education in preventing unintentional injuries among children aged 0–3: randomized controlled trial in Shanghai
Source: BMC Public Health. 2022 Nov 16;22:2086. doi: 10.1186/s12889-022-14462-5 (PMC9666943; doi:10.1186/s12889-022-14462-5)

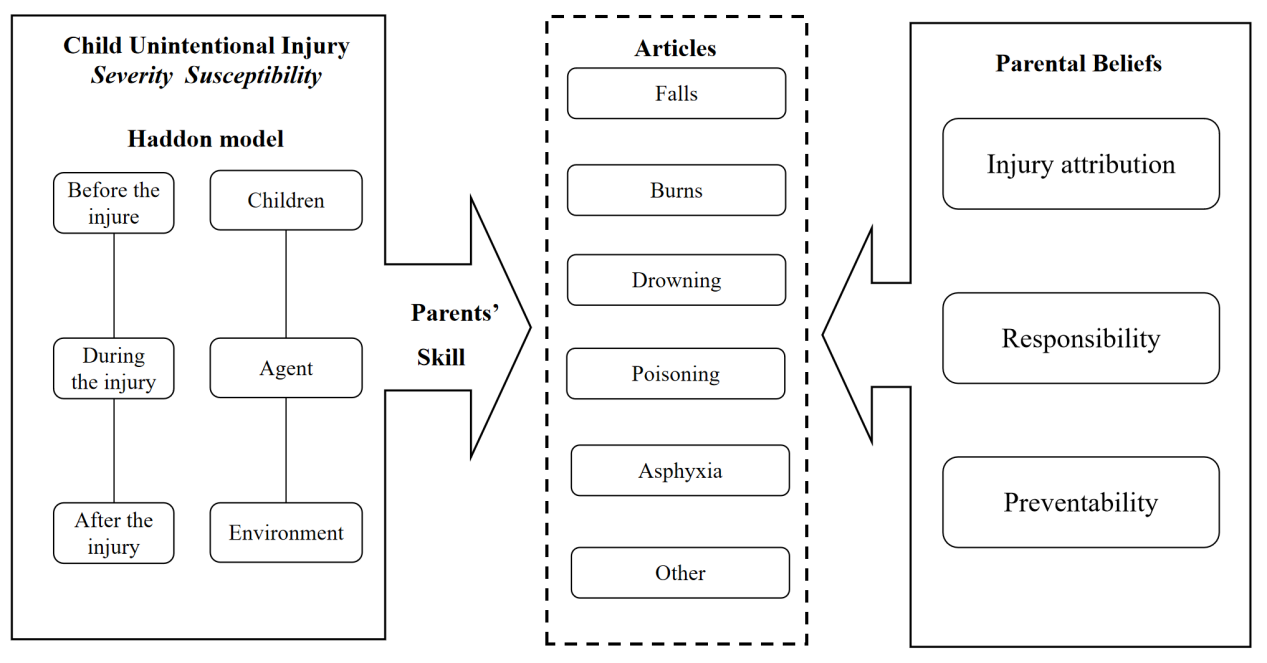


**Figure S1.** Design the articles based on Haddon model and beliefs the parents needed

Supplement: Supplementary file 5 — Additional file 5: Table S2. The details of questionnaire. [file 12889_2022_14462_MOESM5_ESM.docx]

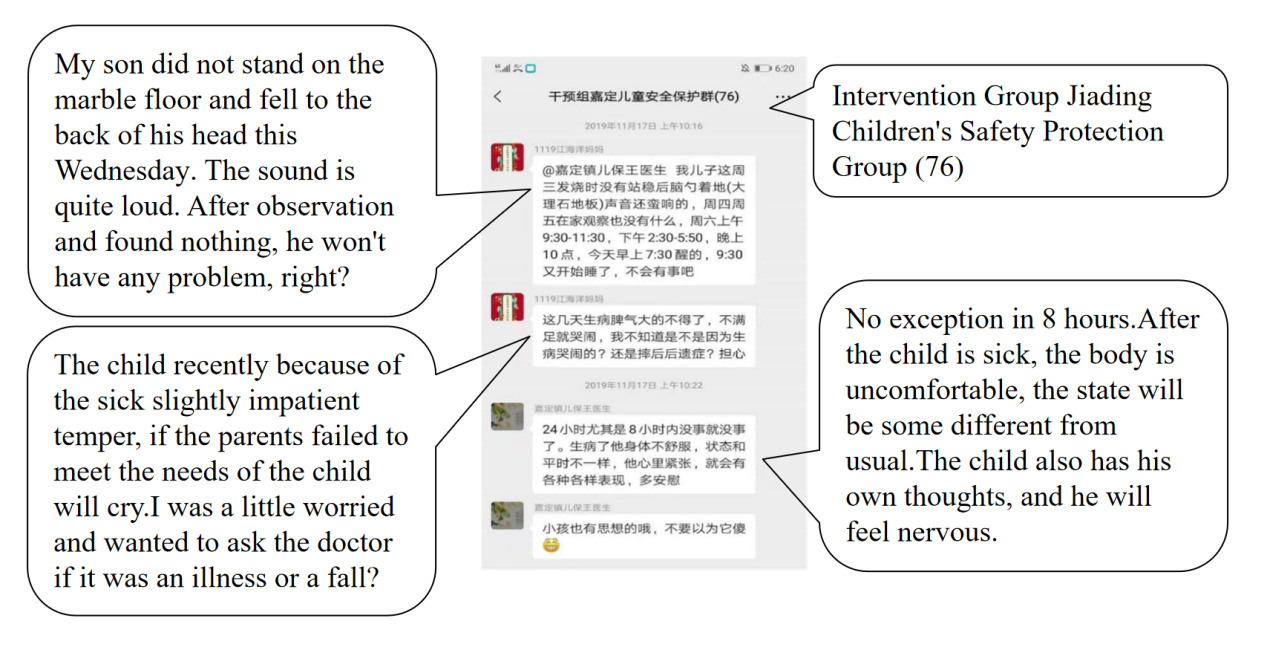


**Figure S3.** Screenshots of parents and doctors communicating with each other in the WeChat group

Supplement: Supplementary file 6 — Additional file 6: Table S3. Acceptance of WeChat among intervention group and control group. [file 12889_2022_14462_MOESM6_ESM.docx]

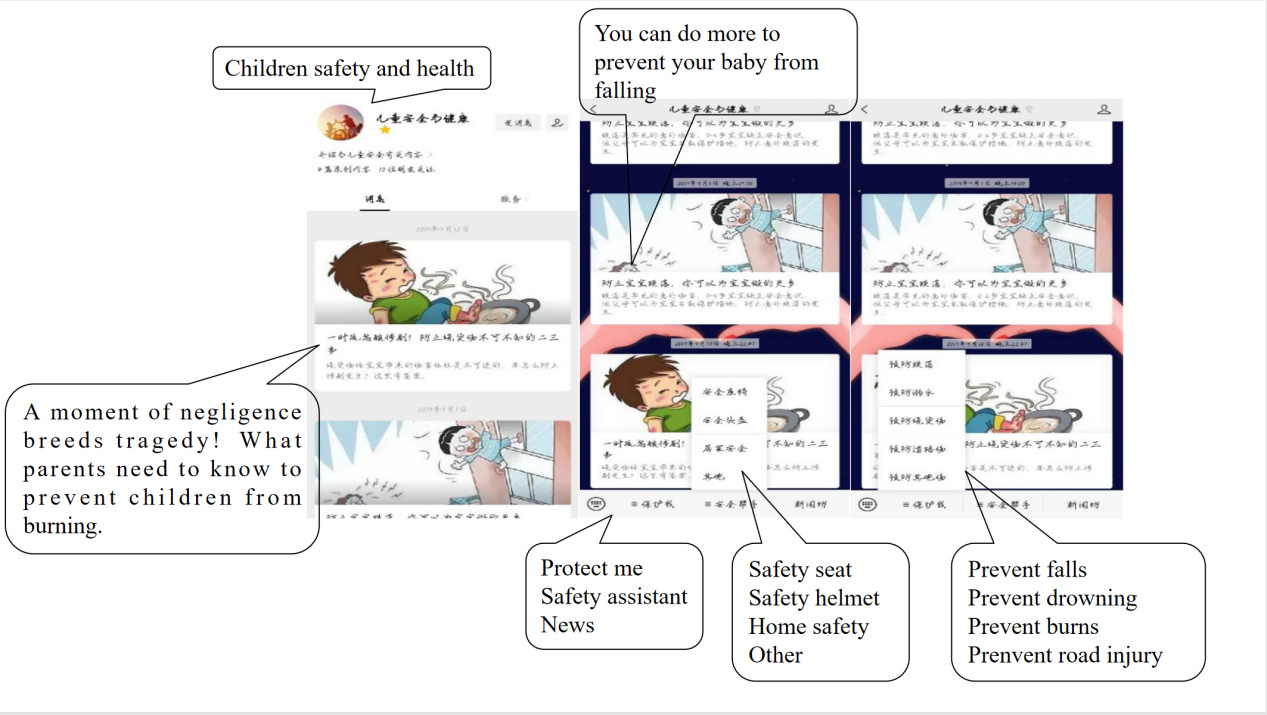


**Figure S2.** Articles uploaded to the WeChat official account

Supplement: Supplementary file 7 — Additional file 7: Table S4. Theeffectiveness of primary and secondary outcome within each group. [file 12889_2022_14462_MOESM7_ESM.docx]
