# Supplementary material for: Effectiveness of WeChat-group-based parental health education in preventing unintentional injuries among children aged 0–3: randomized controlled trial in Shanghai
Source: BMC Public Health. 2022 Nov 16;22:2086. doi: 10.1186/s12889-022-14462-5 (PMC9666943; doi:10.1186/s12889-022-14462-5)
Supplement: Supplementary file 2 [file 12889_2022_14462_MOESM5_ESM.docx]

**Table S2.** The details of questionnaire

| Question | Answer | Score |
| --- | --- | --- |
| Sociodemographic characteristics |  |  |
| 1.What is your relationship with child? | ①Mother, ②Father, ③Other (Investigation terminated) | — |
| 2.How old are you now? | years | — |
| 3.What is your education background? | ①Elementary school, middle school and high school, ②Junior college, ③Undergraduate and above | — |
| 4.What's your occupation? | ①Employees of state-owned enterprises and public institutions, ②Employees of foreign-funded, private and other enterprises, ③ Other (farmers, soldiers, freelancers, etc), ④Unemployed | — |
| 5.What is your annual household income per capital |  | — |
| 6.Do you have any other children in your family besides this child? | ①0, ②1, ③2, ④＞3 | — |
| 7.Child age | years | — |
| 8.Child sex | ①male, ②female | — |
| 9.How many unintentional injuries did your child have in the past year (or since birth)? | ①0, ②1, ③2, ④＞3 | — |
| 10. What types of children injuries have occurred? | ①Falls, ②Burns, ③Suffocation, ④Drowning, ⑤Other (poisoning, animal scratches and bites, etc) | — |
| Knowledge |  |  |
| 1.Children under one year old can sleep on a soft, thick cushion or pillow. | ①True, ②False, ③Unknow | ①True=0, ②False=1, ③Unknow=0 |
| 2.Small toys and plush toys can be placed on the crib. | ①True, ②False, ③Unknow |  |
| 3.Cover small scald areas with ice directly. | ①True, ②False, ③Unknow |  |
| 4.To prevent falls, it is best to use a walker when your child is learning to walk. | ①True, ②False, ③Unknow |  |
| 5.Children will cry out when they are drowning. | ①True, ②False, ③Unknow |  |
| 6.It's impossible for a child to drown at home. | ①True, ②False, ③Unknow |  |
| Skills |  |  |
| 1.What of the following skills do you currently have?(multiple choice) | ①Scald first aid, ②First aid for tracheal foreign body, ③Foreign body swallowing first aid, ④Small wound treatment for first aid, ⑤Selection of Safety seats, ⑥Home Safety skills, ⑦Other          , ⑧No more than | — |
| Beliefs |  |  |
| Injury attribution |  |  |
| 1.It is due to bad luck that children get unintentionally injured. | ①Strongly disagree, ②Disagree, ③Agree, ④Strongly agree | ①Strongly disagree=4, ②Disagree=3, ③Agree=2, ④Strongly agree=1 |
| Responsibility |  |  |
| 1.No matter who takes care of the children, parents have the primary responsibility for their children's safety. | ①Strongly disagree, ②Disagree, ③Agree, ④Strongly agree | ①Strongly disagree=1, ②Disagree=2, ③Agree=3, ④Strongly agree=4 |
| 2.Parents are primarily responsible for helping their children develop an attitude of safety. | ①Strongly disagree, ②Disagree, ③Agree, ④Strongly agree |  |
| Preventability |  |  |
| 1.Unintentional injuries are preventable. | ①Strongly disagree, ②Disagree, ③Agree, ④Strongly agree | ①Strongly disagree=1, ②Disagree=2, ③Agree=3, ④Strongly agree=4 |
| 2.There are many things parents can do to prevent unintentional injuries to their children. | ①Strongly disagree, ②Disagree, ③Agree, ④Strongly agree |  |
| Behaviors |  |  |
| Supervision behaviors |  |  |
| 1.Keeping an eye on the child without letting them out of my sight. | ①Never, ②Rarely, ③Sometimes, ④Often, ⑤Always | ①Never=1, ②Rarely=2, ③Sometimes=3, ④Often=4, ⑤Always=5 |
| 2.I know what the child is doing all the time | ①Never, ②Rarely, ③Sometimes, ④Often, ⑤Always |  |
| 3.Getting people who are not yet adults to help with childcare. | ①Never, ②Rarely, ③Sometimes, ④Often, ⑤Always | ①Never=5, ②Rarely=4, ③Sometimes=3, ④Often=2, ⑤Always=1 |
| 4.When there are other caregivers around, I lower the intensity of child care | ①Never, ②Rarely, ③Sometimes, ④Often, ⑤Always |  |
| 5.Performing other activities, such as playing on the mobile phone, while looking after the child. | ①Never, ②Rarely, ③Sometimes, ④Often, ⑤Always |  |
| Prevent asphyxia behaviors |  |  |
| 1.When my child is crying, I feed him or her. | ①Never, ②Rarely, ③Sometimes, ④Often, ⑤Always | ①Never=5, ②Rarely=4, ③Sometimes=3, ④Often=2, ⑤Always=1 |
| 2.Sleep in the same bed beside my child. | ①Never, ②Rarely, ③Sometimes, ④Often, ⑤Always |  |
| Prevent burning behaviors |  |  |
| 3.When preparing bath water, put hot water first. | ①Never, ②Rarely, ③Sometimes, ④Often, ⑤Always | ①Never=5, ②Rarely=4, ③Sometimes=3, ④Often=2, ⑤Always=1 |
| 4.When I cook, I let my child in the kitchen with me. | ①Never, ②Rarely, ③Sometimes, ④Often, ⑤Always |  |
| Prevent fall behaviors |  |  |
| 5. Install bed guardrail at crib. | ①Never, ②Rarely, ③Sometimes, ④Often, ⑤Always | ①Never=1, ②Rarely=2, ③Sometimes=3, ④Often=4, ⑤Always=5 |
| 6. Make sure the distance among cribs, beds, sofas and other things with windows and balconies are safe. | ①Never, ②Rarely, ③Sometimes, ④Often, ⑤Always |  |
| Prevent drowning behaviors |  |  |
| 7.Empty the water tub immediately after baby is bathed | ①Never, ②Rarely, ③Sometimes, ④Often, ⑤Always | ①Never=1, ②Rarely=2, ③Sometimes=3, ④Often=4, ⑤Always=5 |
| 8.The toilet seat at home is covered | ①Never, ②Rarely, ③Sometimes, ④Often, ⑤Always |  |
