# Supplementary material for: Effectiveness of WeChat-group-based parental health education in preventing unintentional injuries among children aged 0–3: randomized controlled trial in Shanghai
Source: BMC Public Health. 2022 Nov 16;22:2086. doi: 10.1186/s12889-022-14462-5 (PMC9666943; doi:10.1186/s12889-022-14462-5)
Supplement: Supplementary file 3 [file 12889_2022_14462_MOESM7_ESM.docx]

**Table S4.** The effectiveness of primary and secondary outcome within each group

| Variable | Intervention group (n=145) |  |  |  | Control group (n=131) |  |  |  |
| --- | --- | --- | --- | --- | --- | --- | --- | --- |
|  | Baseline (95% CI) | Follow-up (95% CI) | Change (95% CI) | *P* value | Baseline (95% CI) | Follow-up (95% CI) | Change (95% CI) | *P* value |
| Unintentional injury incidence, % | 9.0 (4.1, 13.9) | 11.7 (6.2, 17.2) | 2.8 (-4.8, 10.3) | 0.43 | 9.9 (4.8, 15.0) | 22.9 (15.7, 30.1) | 13.0 (3.7, 22.3) | 0.004 |
| Injury attribution | 3.46 (3.36, 3.55) | 3.59 (3.50, 3.69) | 0.14 (0.04, 0.24) | 0.007 | 3.49 (3.40, 3.58) | 3.38 (3.26, 3.49) | -0.12 (-0.23, -0.01) | 0.04 |
| Responsibility | 6.60 (6.45, 6.76) | 7.52 (7.40, 7.63) | 0.92 (0.75, 1.08) | <0.001 | 6.80 (6.62, 6.98) | 6.99 (6.82, 7.15) | 0.18 (-0.01, 0.38) | 0.07 |
| Preventability | 6.92 (6.75, 7.08) | 7.72 (7.62, 7.81) | 0.80 (0.65, 0.95) | <0.001 | 7.02 (6.83, 7.20) | 6.94 (6.77, 7.11) | -0.08 (-0.23, 0.08) | 0.33 |
| Daily supervision behavior | 16.99 (16.74, 17.24) | 21.88 (21.58, 22.18) | 4.89 (4.51, 5.27) | <0.001 | 17.12 (16.91, 17.33) | 20.75 (19.92, 21.58) | 3.63 (2.77, 4.48) | <0.001 |
| Behaviors of preventing specific injuries | 30.99 (30.37, 31.60) | 36.53 (36.16, 36.91) | 5.55 (5.04, 6.05) | <0.001 | 31.10 (30.49, 31.71) | 32.02 (31.44, 32.61) | 0.92 (0.30, 1.55) | 0.004 |

Abbreviations: 95% CI, 95% confidential interval. OR, odds ratio. The statistical difference compared intervention group and control group was argued by 95% CI whether contained “0”.

The 95% CI was calculated by follow-up minus baseline.

P value is the result of comparing baseline with follow-up data in intervention group and control group.
